# Thermal Degradation Mechanism of a Thermostable Polyester Stabilized with an Open-Cage Oligomeric Silsesquioxane

**DOI:** 10.3390/ma11010022

**Published:** 2017-12-24

**Authors:** Yolanda Bautista, Ana Gozalbo, Sergio Mestre, Vicente Sanz

**Affiliations:** University Institute of Ceramic Technology, Jaume I University, 12006 Castellón, Spain; ana.gozalbo@itc.uji.es (A.G.); sergio.mestre@itc.uji.es (S.M.); sanzs@qui.uji.es (V.S.)

**Keywords:** oligomeric silsesquioxane, thermal degradation, open cage structure, thermostable polyester, FTIR volatiles analysis

## Abstract

A polyester composite was prepared through the polymerization of an unsaturated ester resin with styrene and an open-cage oligomeric silsesquioxane with methacrylate groups. The effect of the open-cage oligomeric silsesquioxane on the thermal stability of the thermostable polyester was studied using both thermogravimetric analysis and differential thermal analysis. The results showed that the methacryl oligomeric silsesquioxane improved the thermal stability of the polyester. The decomposition mechanism of the polyester/oligomer silsesquioxane composite was proposed by Fourier transform infrared spectroscopy (FTIR) analysis of the volatiles.

## 1. Introduction

Unsaturated polyester resins are the most frequently used organic matrices in composite materials. Therefore, their use has extended to numerous fields such as the maritime, automotive, and aeronautical transport sectors, the energy field (in the wind turbine industry), or the building sector, and in industries manufacturing panels, bathroom components, or electrical wiring [1]. Unfortunately, the polyesters prepared with these resins are flammable materials with spontaneous flame propagation in the presence of oxygen.

The high cost in human lives and material damage that can result from a fire has greatly promoted the development of flame retardants. These products usually act by suppressing or delaying the physical-chemical processes that occur during fire by eliminating some of the components of the fire cycle [2,3].

The nature of the flame retardants used commercially in polyesters varies greatly. Originally, halogenated compounds were used, but they were soon removed due to the toxicity of the gases generated in their decomposition. Subsequently, aluminum trihydroxide (ATH) was introduced, thus avoiding the toxicity of the gases, but with the disadvantage of a very low consistency of the polymer ashes after fire. Recently, considerable attention has been paid to the effect of silsesquioxane, usually in cage structures, on a polymer’s thermal resistance [4]. An improvement has been found in epoxy [5,6], polycarbonate [7,8], polystyrene [9], polyurethane [10], and polymethyl methacrylate [11] polymers.

In previous work [12,13], our research group improved both the thermal and fire behavior of a polyester by adding silsesquioxane oligomers together with ATH. The LOI (limiting oxygen index) value was increased from 21% for the original polyester to 51% for the samples to which ATH and silsesquioxane were simultaneously added. It was also observed that the incorporation of an open-cage silsesquioxane to the mixture of polyester and ATH produced a fourfold increase in the mechanical resistance of the ashes. In contrast, there was no increase in mechanical resistance when a close-cage form of the silsesquioxane was added instead.

In this work, the effect of the open-cage oligomeric silsesquioxane on the thermal stability of the polyester was studied by using both thermogravimetric and differential thermal analysis, with a simultaneous analysis of the volatiles by Fourier transform infrared spectroscopy (FTIR). A thermal decomposition mechanism was proposed for the polyester/oligomer open-cage silsesquioxane composite. Although the thermal degradation of polyesters has been extensively studied over the last thirty years [14,15,16,17,18,19,20,21,22,23,24,25], the effect of open-cage silsesquioxane has yet to be assessed. Even though the analyzed polyesters were quite different, the same three general processes were found: degradation of the main chain of the polyester, degradation of the styrene interconnections, and oxidation of the products generated in both of those previous ones. However, the temperature at which every process proceeded, as well as the type of products generated, were significantly different, depending on several factors such as the type of glycol, the nature of the aromatic or the saturated diacids, the initiator, or the curing process [15]. 

## 2. Results and Discussion

**Figure 1:**
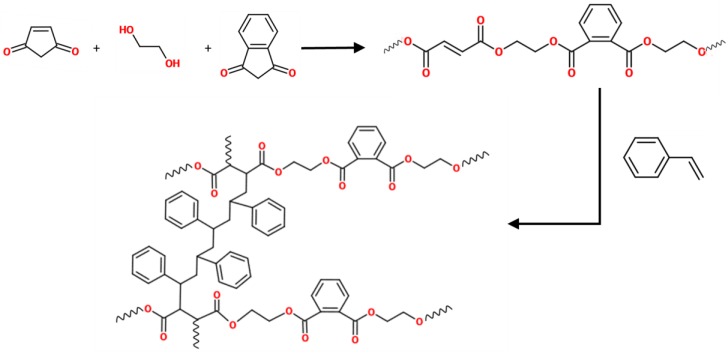
Scheme of the synthesis and polymerization of an unsaturated polyester resin.

A commercial unsaturated polyester resin was obtained by the reaction between maleic anhydride, phthalic anhydride, and ethylene glycol. Then, the main chain of the resin had ester groups, aromatic rings, and unsaturated sites, as shown in Figure 1. Usually, polyester resins are mixed with styrene, which acts as both a diluent and as a cross-linking agent. Polymerization involves the reaction of the unsaturated sites in the resin with the vinyl group of the styrene, as shown in Figure 1.

**Figure 2:**
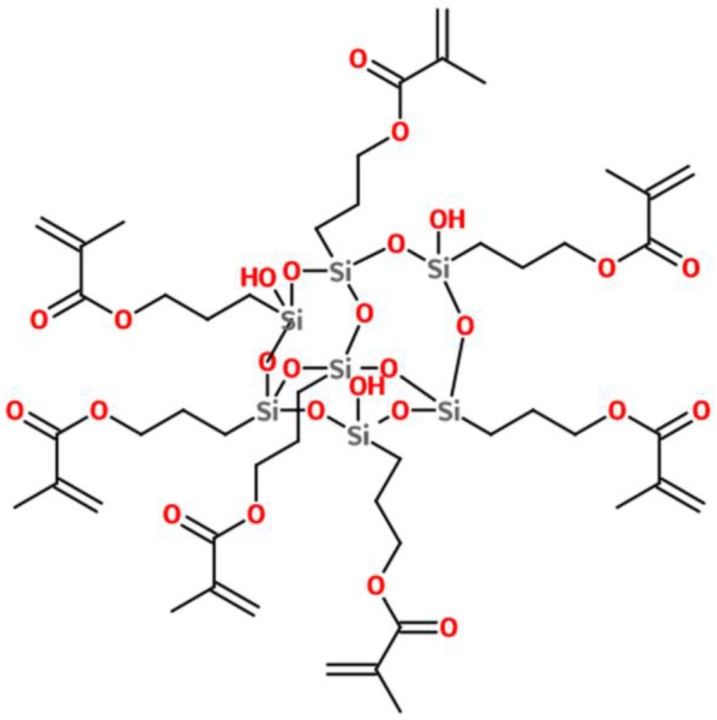
Scheme of one of the molecules of the organic-inorganic hybrid oligomer [12].

The organic–inorganic hybrid oligomer was synthesized by the sol–gel method from 3-methacryloxypropyltrimethoxysilane (MAPTMS) with hydrolyzable methoxy groups, which allowed the formation of siloxane bonds. The oligomer consisted mainly of mixtures of four to eight silsesquioxane molecules containing unreacted hydroxyl groups (hydrolyzed silanes, but not yet condensated) [12]. The participation of these groups in hydrogen bonds with the methacrylate groups prevented the condensation reactions from concluding. The oligomer exhibited a high number of cycles per molecule without forming the closed polyhedral structures characteristic of POSS (polyhedral oligomeric silsesquioxane). Figure 2 details an example of one of the molecules that formed the oligomer.

### 2.1. Thermal Degradation of the Thermostable Polyester

The thermal degradation of thermostable polyesters is a complex, heterogeneous process of several exo/endothermic reactions producing volatile compounds. The weight loss of the sample was registered by thermogravimetric analysis, and the volatiles were identified by infrared spectroscopy. Differential thermal analysis (DTA) and differential scanning calorimetry (DSC) were used to follow the heat generated.

**Figure 3:**
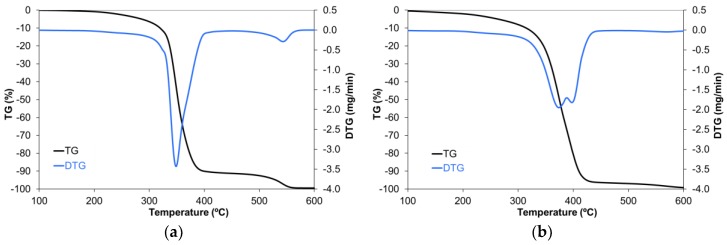
Thermogravimetric analysis of the thermostable polyester (**a**) in air; and (**b**) in nitrogen.

Thermogravimetric analysis can provide information not only on the thermal stability, but also on the reactions taking place. Figure 3 shows the thermogravimetric analysis of the thermostable polyester in (a) air, and (b) nitrogen atmospheres. In air (Figure 3a), two groups of chemical transformations with associated mass loss could be appreciated. The first group took place between 200 °C and 450 °C and accounted for 90% of the total mass loss. The asymmetric shape of the peak suggested that it was the result of several steps. The second group was registered between 450 °C and 570 °C, and accounted for the remaining mass loss. As this last peak was only observed when thermal degradation occurred in the presence of oxygen, it could correspond to a combustion process [25].

**Figure 4:**
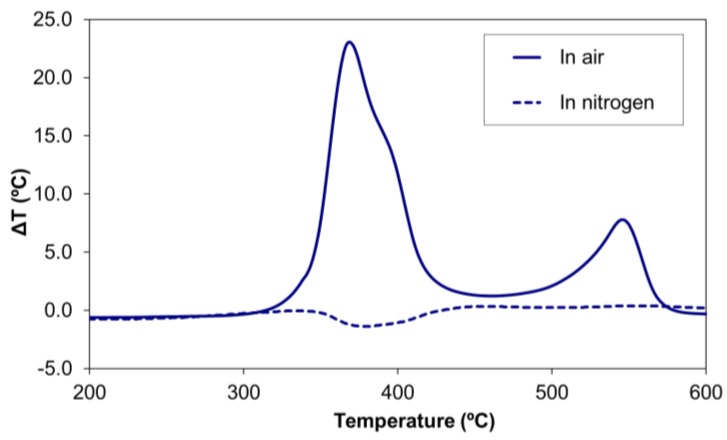
Differential thermal analysis (DTA) of the thermostable polyester in air and nitrogen atmospheres (heating rate of 15 °C/min).

The differential thermal analysis (Figure 4) showed that the group of chemical transformations occurring between 200 °C and 450 °C in the absence of oxygen were slightly endothermic processes, which would likely correspond to pyrolytic reactions [14]. When the thermal degradation was studied in the air atmosphere, both groups of chemical transformations were highly exothermic, raising the temperature as much as 23 °C for a polymer sample of 15 mg. Although pyrolytic reactions also occur in an air atmosphere, they are masked by the highly exothermic combustion reactions of the volatiles [26]. The higher-temperature chemical transformations (450–570 °C) were exothermic combustion processes.

**Figure 5:**
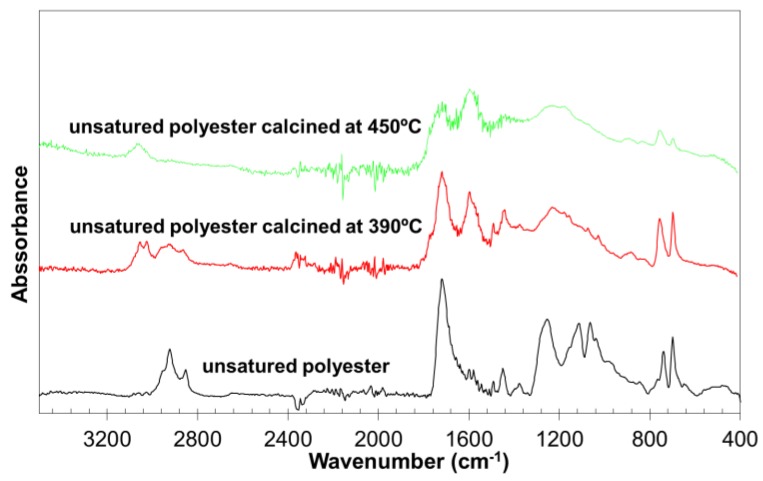
Fourier transform infrared (FTIR) spectra of the original thermostable polyester and the ashes generated after calcination at 390 °C and 450 °C.

Figure 5 compares the infrared spectra of the polyester calcined at two different temperatures with that of the original polyester. A decrease of the signals related to the ester groups (1720, 1320, and 940 cm^−1^) and the aliphatic groups (2930, 2860, and 1450 cm^−1^) was observed, whereas the signals of the aromatic groups (3060, 3030, 1600, 760, and 700 cm^−1^) increased. Therefore, the thermal degradation of the polyester generated ashes was enriched in aromatic groups by the formation of polyphenolic structures [14].

**Figure 6:**
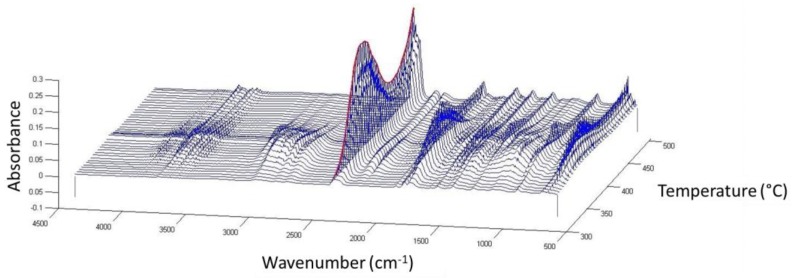
FTIR spectra of the volatile species produced during the thermal degradation of the polyester as a function of temperature, in air atmosphere.

**Figure 7:**
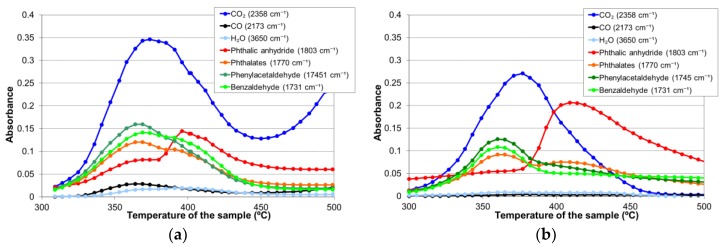
Evolution with temperature of the infrared signal of each identified volatile species during the thermal degradation of the polyester (**a**) in air atmosphere; and (**b**) in nitrogen.

To study the thermal degradation of the thermostable polyester, FTIR spectra of the sample volatiles were taken at each 5 °C for calcination temperatures ranging from 300 °C to 500 °C (Figure 6). Table 1 enumerates the species corresponding to each of the main infrared peaks found. The evolution of each infrared signal with temperature (giving a semi-quantitative information about the volatile species) is depicted in Figure 7, for calcination in air and nitrogen atmospheres. 

As can be seen in Figure 7, the thermal degradation of the polyester in both atmospheres produced two groups of volatile species between 300 °C and 450 °C. The first group appeared around 365 °C and corresponded mainly to species such as carbon dioxide, aldehydes, and phthalates, while the second one, appearing between 400 °C and 450 °C, essentially consisted of phthalic anhydride. Carbon dioxide was registered in both the air and nitrogen atmospheres; thus, it can be considered as a product of both pyrolysis and combustion reactions. Carbon monoxide and water were also detected as reaction products in both atmospheres, though their proportion was practically negligible when the assay was performed in nitrogen.

### 2.2. Thermal Degradation of the Polyester/Oligomer Silsesquioxane Composite

As shown in a previous work [12], the silsesquioxane oligomer synthetized was a mixture of compounds obtained by condensation of between four to eight silane molecules, with a high number of intramolecular cycles and the complete hydrolysis of the alkoxide groups. The presence of hydrogen bonds between the carbonyl groups of the methacrylate and the hydroxyls of the hydrolyzed alkoxides prevented the condensation reactions from concluding, so open-cage structures of the polyhedral oligomer silsesquioxanes were obtained. These open-cage structures were selected because their ashes showed better mechanical resistance [12]. Next, 5 wt % of this open-cage oligomer silsesquioxane was incorporated into the mixture of unsaturated polyester resin and styrene prior to polymerization to obtain the polyester/oligomer silsesquioxane composite studied below.

**Figure 8:**
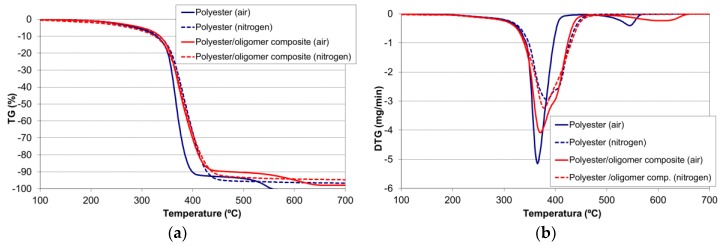
Thermogravimetric analysis (—) in air atmosphere and (---) in nitrogen, at 15 °C/min for the polyester and the polyester oligomeric silsesquioxane composite: (**a**) thermogravimetry (TG); and (**b**) differential thermogravimetry (DTG).

The thermal degradation of the polyester/oligomer silsesquioxane composite was studied by the same characterization techniques previously used for the polyester. Thermogravimetric analysis (Figure 8) showed a slight decrease in both the decomposition rate and weight loss. The thermal degradation processes were delayed to higher temperatures. For example, in the air atmosphere at 400 °C, the thermostable polyester exhibited a weight loss of 90%, whereas that of the composite material amounted only to 60%; the thermo-oxidative process that took place at 540 °C for the polyester was observed at 600 °C for the composite material.

**Figure 9:**
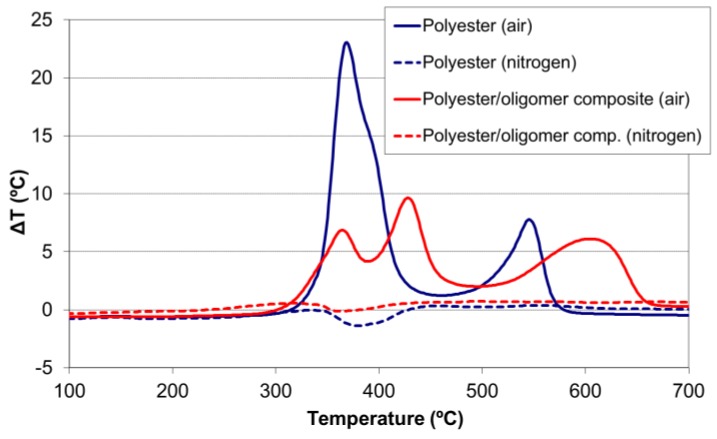
Differential thermal analysis (DTA) of thermostable polyester and polyester/oligomer silsesquioxane composite, in air atmosphere (—) and in nitrogen (---) (at 15 °C/min).

A reduction in the exothermicity of the thermal degradation reactions was also observed in the polyester/oligomer silsesquioxane composite (Figure 9) due to these two effects: the decrease in the rate of the first reaction (which peaked at 365 °C), and the shift of the other degradation processes (those with peaks at 390 °C and 540 °C) to higher temperatures (450 °C and 600 °C, respectively). 

**Figure 10:**
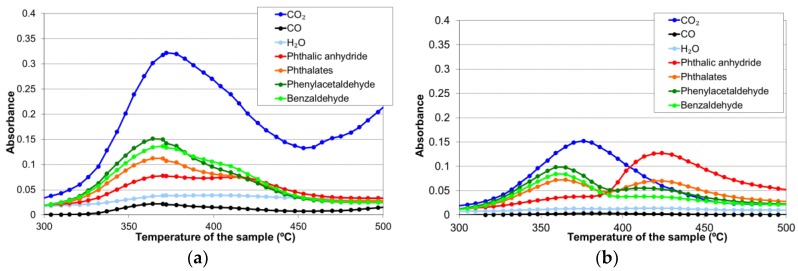
The evolution with the temperature of the infrared signal of each identified volatile species during the thermal degradation of the polyester/oligomer silsesquioxane composite (**a**) in the air atmosphere; and (**b**) in nitrogen.

The volatile species found in the thermal degradation of the polyester/oligomer silsesquioxane composite were of the same nature as those found in the decomposition of the thermostable polyester, with only slight changes in relative intensities with temperature (Figure 10).

### 2.3. Mechanism for the Thermal Degradation of the Thermostable Polyester and the Composite

To evaluate the pyrolytic decomposition mechanism, both the identified volatile species and the information included in the literature were considered. Comparison with the decomposition products of polystyrene and polyester allowed us to identify which part of the polymer (main chain or chain-interconnections) gave rise to which degradation product.

#### 2.3.1. Aldehyde Generation

The main decomposition products of the polystyrene identified in the literature were toluene, ethylbenzene, styrene, α-methylbenzene, benzaldehyde, and phenylacetaldehyde. In this study, only the aldehydes were identified (see Table 1). These gases were mainly produced in the process taking place around 365 °C in both atmospheric conditions. 

Anderson and Freeman [27] described the chemical mechanism of benzaldehyde formation by the thermal degradation of styrenated polyester in the presence of oxygen. These authors proposed the attack of the oxygen molecule on the carbon in the α position of the phenyl group, with the formation of an unstable hydroperoxide intermediate. Then, the bonds ruptured and rearranged by transferring the hydrogen atom of the carbon in the β position to the benzoyl radical. This mechanism was also applied to the thermo-oxidative degradation of polystyrene in the presence of oxygen [28] but, unfortunately, could not be used to explain the formation of those aldehydes when no oxygen is present.

**Figure 11:**
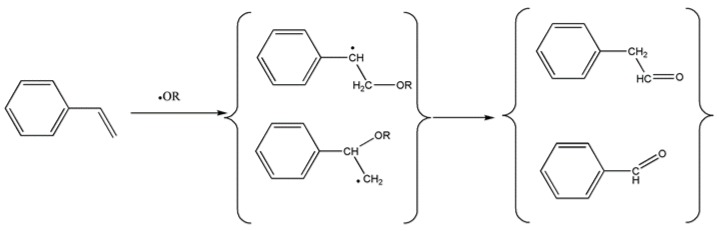
Chemical mechanism proposed by Evans for the formation of both aldehydes in the presence of oxygenated free radicals [20].

In the present study, both aldehydes were generated simultaneously. Evans et al. [20] explained the simultaneous generation of these aldehydes by the presence of oxygenated free radicals, formed in the initial radical steps. The addition of the radical to the carbon in the α or β position to the phenyl group produced either one or the other aldehyde (Figure 11). This mechanism could explain the simultaneous presence of both aldehydes, not only in air, but also in the nitrogen atmosphere, for the thermal decomposition process that took place around 365 °C.

#### 2.3.2. Phthalic Anhydride Formation

Phthalic anhydride was mainly produced in the decomposition process that occurred around 390 °C. Ravey [29] explained the formation of this molecule through a mechanism that started with the elimination of the phthalic anhydride directly from the main chain of the polyester, and proceeded with the recombination of the two radicals to obtain an ether group. If this were the case, 3-phenyl-2-propenoic acid or even diethylene glycol should have been detected, in addition to phthalic anhydride. As neither of these compounds were found in the volatiles, Ravey’s mechanism was not considered for the thermal decomposition of the composite.

The mechanism proposed by Sivasamy [30] was based on a β-elimination process. Hydrogens in the β position to the benzoyl radical could contribute a cyclic six-center transition state. According to this mechanism, the acid would first be obtained, and then dehydration would occur, leading to the anhydride. However, as no acid volatile compounds were detected by FTIR spectroscopy, this mechanism was also discarded. 

**Figure 12:**
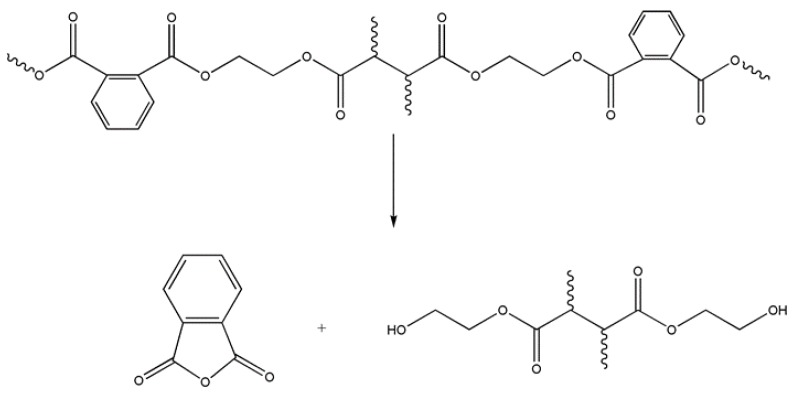
Mechanism proposed by Anderson and Freeman to explain the formation of phthalic anhydride [27].

**Figure 13:**
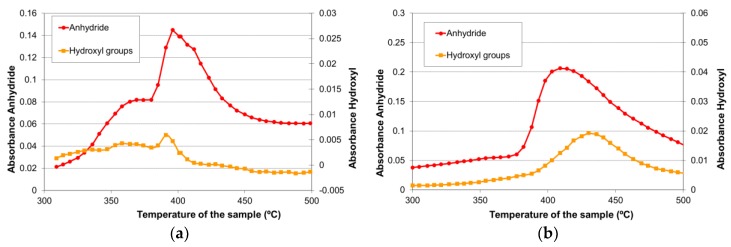
The evolution with the temperature of the infrared signal of the phthalic anhydride and the hydroxyls groups in the thermal decomposition of the polyester (**a**) in the air atmosphere; and (**b**) in the nitrogen atmosphere.

Anderson and Freeman [27] proposed a different mechanism for the generation of phthalic anhydride in the thermal decomposition process of the polyester (Figure 12). The reaction started with the homolytic cleavage of the bond closer to the ester group and the formation of free radicals. Later, reorganization of the bonds would produce phthalic anhydride and hydroxyester compounds. Figure 13 shows that hydroxyl groups were detected simultaneously to phthalic anhydride in the FTIR spectroscopic characterization, so the Anderson and Freeman mechanism could explain the second pyrolysis stage of the thermal decomposition process of the polyester main chain that started around 390 °C in both atmospheric conditions. These FTIR signals corresponding to the hydroxyls vibrations have been shown with expanded axes in Figure 13, but were not included in Figure 7 and Figure 10 due to their low value. 

#### 2.3.3. Main Chain Scission

Carbon dioxide was detected in the thermal decomposition of the studied polyester and of the composite in both atmospheric conditions. In the air atmosphere, a pyrolytic reaction was followed by the occurrence of the consecutive combustion of the volatile species, with carbon dioxide as the main product. However, in the nitrogen atmosphere, carbon dioxide must be generated directly by the pyrolysis reactions.

Several studies have been found in the literature that describe the thermal decomposition process of polyester, with carbon dioxide as the main reaction product [31,32,33,34,35]. Bikiaris et al. [35] proposed that the thermal degradation of an aliphatic polyester in a nitrogen atmosphere proceeded through the decomposition of the carboxylic groups in terminal positions of the polyester chains.

Pohl [31] studied the effect of the chemical structure on the thermal stability of polymers and concluded that the presence of ester groups in the polymer main chain considerably reduced the thermal stability of the polymer. Therefore, he proposed that the degradation process took place by breaking bonds closer to the ester groups and producing small volatile molecules. Zimmermann [33] also described the starting degradation steps of polyesters as random scissions of the polymer chain by the ester unions. Baudry [18] even described these scission products as alkyl or alkoxy radicals. 

#### 2.3.4. Summary

Considering the species that are identified, the thermal decomposition of this thermostable polyester in the interval 300–500 °C can be described as follows.

**Figure 14:**
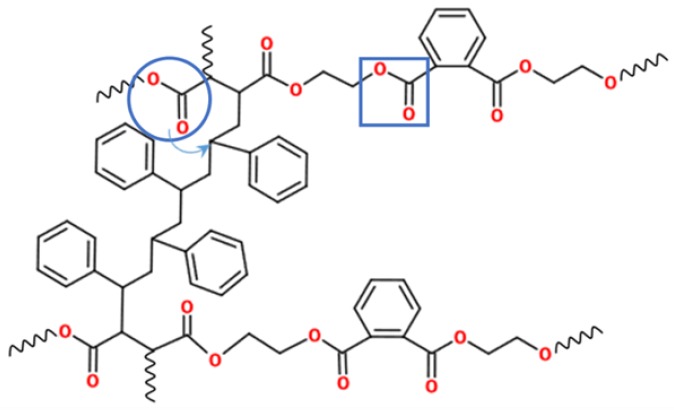
Chemical structure of the studied polyester. The two different ester groups are marked.

From a thermal point of view, the weakest points in the polyester material are the ester groups. In the studied thermostable polyester, there are two types of ester groups (Figure 14): those in the aliphatic chains that come from the maleic anhydride (marked with a circle), and those closer to the aromatic rings that come from the phthalic anhydride (marked in the figure with a square). The scission of the polymer chain by both ester unions generates reactive alkoxy radicals as the reaction product, as described by Pohl, Zimmermann, and Baudry.

The second type of ester groups are stabilized by their proximity to the aromatic ring, so that the ester groups in the aliphatic chains break at lower temperatures (365 °C), generating oxygenated free radicals. These reactive radicals, which are close to the styrened chain-interconnections, simultaneously produce benzaldehyde and phenylacetaldehyde by the radical mechanism postulated by Evans.

The ester groups closer to the aromatic rings break at higher temperatures (390 °C), generating phthalic anhydride by the mechanism postulated by Anderson and Freeman.

In the present study, those differences in behavior between the ester group were observed, either in the infrared analysis of the volatile species (Figure 7 and Figure 10) or in the DTA (Figure 9). In the case of the DTA experiments in air, the original polyester showed a single exothermic peak that was the result of two overlapping pyrolysis-combustion processes, corresponding to both types of ester groups. This single exothermic peak split in two for the composite, due to a stabilization of the ester groups marked with a square (by interaction with the oligomer) that shifted the second pyrolysis-combustion process 25 °C to higher temperatures. Such an increase in the temperature of thermal decomposition of a polydimethylsiloxane (PDMS) polymer by a reaction with a close-cage POSS oligomer with vinyl groups was reported in the literature by Yang et al. [36]. Only when these silsesquioxane oligomers were chemically incorporated was the thermal stability improved. 

In this work, a certain stabilization in the ester group of the aliphatic chains was also observed for the composite. Although their pyrolysis process occurred at the same temperature as that of the polyester sample (365 °C), the slight decrease in the heat released indicated there was a slowdown of the decomposition rate for the composite. A similar decrease in the heat released was found in the second process. The two effects resulted in a lower thermal degradation of the composite.

## 3. Materials and Methods

### 3.1. Materials

3-Methacryloxypropyltrimethoxysilane (MAPTMS) supplied by Sigma-Aldrich Química, (Madrid, Spain) S.A., was selected as the precursor of the silsesquioxane oligomer, for it has reactive unsaturations in the methacrylate group. Unsaturated polyester resin (obtained by reaction between maleic anhydride, phthalic anhydride, and ethylene glycol, and later diluted in styrene) was kindly supplied by Ferro Spain, (Almassora, Castellon, Spain) S.A. Accelerator NL-51P, a cobalt(II) 2-ethylhexanoate, 6 wt % Co in solvent, was employed as an accelerator, and Butanox LA, methyl ethyl ketone peroxide (MEKP) solution, as an initiator. Both additives were supplied by AzkoNobel (Barcelona, Spain).

### 3.2. Preparation of the Silsesquioxane Oligomer

Synthesis was performed under acid-catalyzed conditions. In a typical synthesis, 20 g (0.08 mole) of MAPTMS were poured into a 200 mL container, and 6.5 g (0.36 mole) of acidified water (HCl 0.01 M) was slowly added while the silane was stirred. Then, the synthesis was performed with a H_2_O:Si mole ratio = 4.5:1 with constantly stirring for 264 h. The characterization of the obtained product is described in detail in a previous paper [12].

### 3.3. Preparation of the Thermostable Polyester

Unsaturated polyester resin was polymerized with 1 wt % of the accelerator NL-51P and 2 wt % of the initiator Butanox LA. The methyl ethyl ketone peroxide (MEKP) was selected as the initiator instead of benzoyl peroxide owing to the fact that the polyesters obtained were more stable [37]. The mixture was poured into molds before gel time to obtain samples with the dimensions 20 mm × 80 mm × 3 mm.

### 3.4. Preparation of the Polyester/Oligomer Silsesquioxane Composite

The polyester/oligomer silsesquioxane composite was prepared as the polyester, but by adding 5 wt % of silsesquioxane oligomer prior to adding the initiator and pouring into the molds. Samples of similar dimensions to the polyester were obtained.

### 3.5. Sample Characterization

The Fourier transform infrared spectroscopy (FTIR) spectra of the samples were obtained with a NICOLET 6700 THERMO spectrometer (Thermo Fischer Scientific Inc., Waltham, MA, USA), at an average of 16 scans at a resolution of 4 cm^−1^. The thermal gravimetric analysis (TGA) and differential thermal analysis (DTA) were performed on a TGA/SDTA 851e (Mettler Toledo International Inc., L’Hospitalet de Llobregat, Spain), by placing cylindrical samples (5 mm diameter, 0.5 mm thickness) in the platinum crucible and heating up to 700 °C at a heating rate of 15 °C/min, in both flowing nitrogen and static air atmospheres. The differential scanning calorimetry (DSC) measurements were performed on a STA 449 C JUPITER (Netzsch, Sant Joan Despí, Barcelona, Spain) instrument. The volatiles were characterized by infrared spectroscopy with a coupled TGA-IR to the DSC instrument.

## 4. Conclusions

Polyester was prepared by the polymerization of an unsaturated ester resin with styrene. An open-cage silsesquioxane with methacrylate groups was employed in the preparation of the polyester/oligomer silsesquioxane composite to improve the thermal behavior of the polymer. Thermogravimetric analysis combined with infrared spectroscopy has been shown to be a powerful technique in the thermal decomposition studies of polymers, even though previous knowledge of the original chemical structure of the polymeric system is needed to propose a pyrolysis mechanism.

The thermal degradation of the thermostable polyester and that of the polyester/oligomer silsesquioxane composite are complex, heterogeneous processes of several reactions that depend on temperature and atmosphere. Between 300 °C and 500 °C, two groups of chemical transformations were observed, with scission of the polymer chains by both ester unions. These processes likely resulted in the generation of reactive radicals, as the literature describes.

The scission of the polymer chain by the ester groups in the aliphatic chains occurred around 365 °C, and those reactive radicals close to the styrened chain-interconnections simultaneously generated benzaldehyde and phenylacetaldehyde by the radical mechanism postulated by Evans et al. [20].

The scission of the polymer chain by the ester groups closer to the aromatic rings, which was observed around 390 °C for the polyester, produced phthalic anhydride by the mechanism postulated by Anderson and Freeman [27].

The presence of 5 wt % open-cage oligomeric silsesquioxanes in the polyester composite affected both pyrolytic transformations: there was a slight decrease in the decomposition rate of the first process at 365 °C, and a displacement of the second pyrolytic process to higher temperatures; this second effect was due to a stabilization of the ester groups closer to the aromatic rings. Both effects contributed to a more gradual release of heat in the combustion reactions, thus decreasing the rate of thermal degradation and improving the fire resistance of the polyester composite.

## Figures and Tables

**Table 1:** Infrared signals of the volatile species identified in the thermal degradation of the polyester.

Wavenumber (cm^−1^)	Identified Volatile Species
3720, 3620, 2358, 670	CO_2_
2173, 2080	CO
3650, 1650, 1490	H_2_O
1770, 1290, 1130	Phthalates
3090, 2810, 2730, 1731	Benzaldehyde
3070, 2710, 1745, 1030	Phenylacetaldehyde
1803, 1250, 890	Phthalic anhydride
